# Risperidone-related bilateral cystoid macular edema: a case report

**DOI:** 10.1186/s13256-019-1978-y

**Published:** 2019-03-13

**Authors:** Anna Kozlova, Charles D. McCanna, Rony Gelman

**Affiliations:** 1 Department of Ophthalmology, State University of New York, Downstate Medical Center, 450 Clarkson Ave, Brooklyn, NY 11203 USA; 2 Downstate College of Medicine, State University of New York, Brooklyn, NY USA

**Keywords:** Cystoid macular edema, Risperidone, OCT, Fluorescein angiography, Case report

## Abstract

**Background:**

A report of the second known case of bilateral cystoid macular edema in a patient taking risperidone.

**Case presentation:**

We report a case of a 69-year-old African American woman using risperidone who presented with worsening visual acuity and was found to have bilateral cystoid macular edema. Upon decreasing the dosage of risperidone, the cystoid macular edema resolved and visual acuity markedly improved. Fluorescein angiography and optical coherence tomography were used to document the severity of cystoid macular edema and subsequent resolution after decreased dosage of risperidone.

**Conclusion:**

The documentation of a patient who developed cystoid macular edema associated with risperidone usage indicates that it may be beneficial to monitor patients taking risperidone for the development of maculopathy.

## Background

Cystoid macular edema (CME) develops with the accumulation of fluid in the macula, causing blurred or diminished central vision. It has a broad differential diagnosis that includes surgical, vascular, structural, and medication-related causes [1].

The retina is vulnerable to medication-induced changes through a variety of mechanisms, although, ultimately, CME develops when the capillary filtration rate is greater than the rate of fluid removal by glial and retinal pigment epithelium (RPE) cells. Although mechanisms have not been proven, some medications are associated with CME. E2 prostaglandins can disrupt the tight junctions of retinal capillaries causing CME. Niacin, in a dose-dependent manner, can also result in CME [2].

In 2013, the first case of risperidone-related bilateral CME was reported by Manousaridis and Gupta [3]. They described a 65-year-old woman with a past medical history of depression who presented with a 5-week history of bilateral blurred vision. Bilateral CME was noted and confirmed with fluorescein angiography (FA) and optical coherence tomography (OCT). The CME resolved with drug removal, with “probable” likelihood of the effect being an adverse drug reaction [4]. Here we describe the second known case of risperidone-associated bilateral CME in the medical literature.

## Case presentation

Our patient is a 69-year-old African American woman who presented with gradually decreased and blurred vision of approximately 1 year’s duration without other ocular symptoms. Her past medical history was significant for hypertension, schizophrenia, and depression with no history of diabetes. Her past ocular history was significant for: uncomplicated cataract extraction of both eyes 2 years prior; primary open-angle glaucoma treated with latanoprost, brimonidine, and timolol in both eyes; and dry eye syndrome with past punctal plug placement. Medications included citalopram, risperidone, amlodipine, enalapril, and metoprolol. She reported no difficulty with medication compliance. Of note, an eye examination approximately 1 year prior to presentation showed 20/20 visual acuity bilaterally. A chart review revealed that she had been taking risperidone 2 mg/day for at least 3 years prior to presentation. Her dosage was increased by her psychiatrist 2 years prior to presentation to 3 mg/day, with ocular symptoms developing approximately 1 year after the dosage increase (or 1 year prior to presentation).

**Fig. 1:**
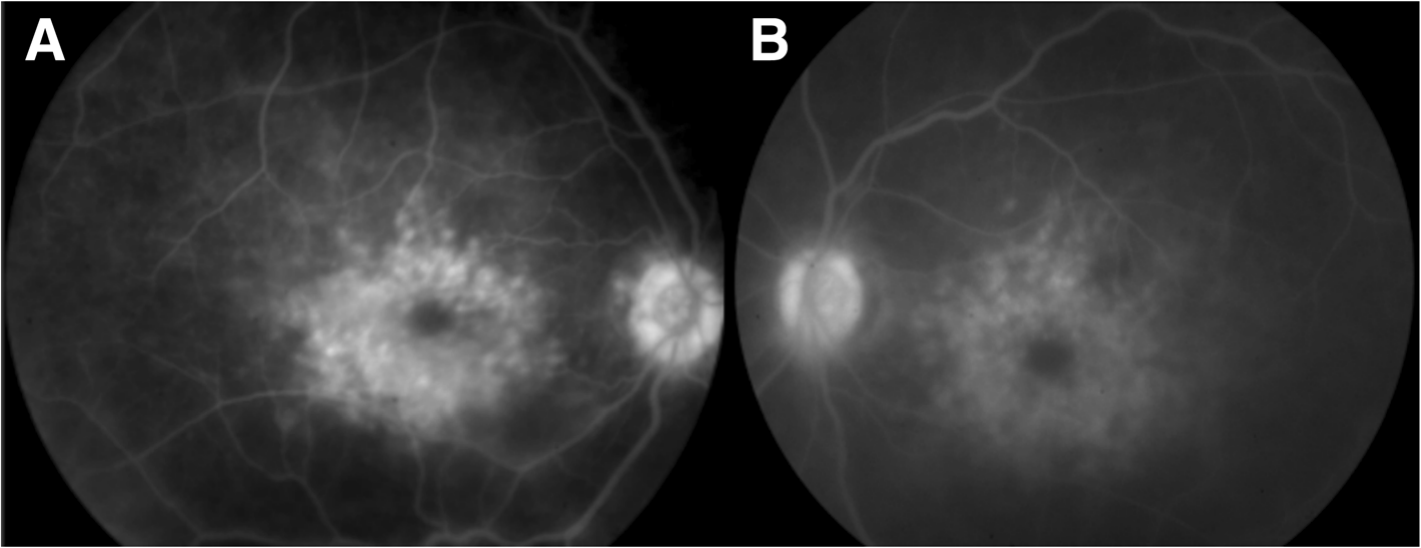
Fluorescein angiography of the right (**a**) and left (**b**) eye showing late petaloid leakage with a hot nerve in both eyes

**Fig. 2:**
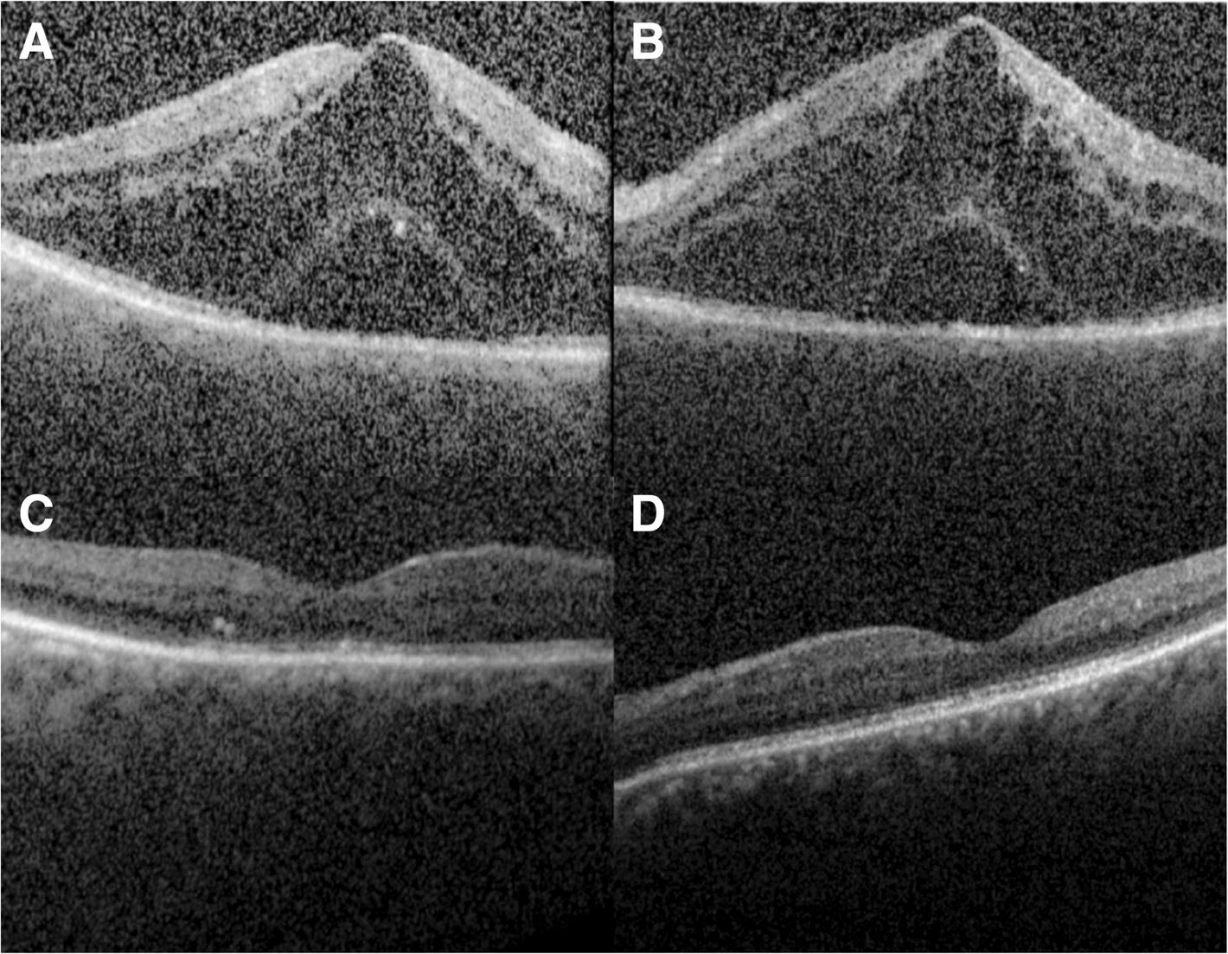
Spectral-domain optical coherence tomography horizontal line scans through the fovea showing cystoid macular edema and subretinal fluid in (**a**) right eye and (**b**) left eye on presentation. Spectral-domain optical coherence tomography following decreased dosing of risperidone showing resolved cystoid macular edema and subretinal fluid in the (**c**) right eye and (**d**) left eye at 4-months follow-up

Visual acuity on presentation was 20/150 in her right eye and 20/200 in her left eye and intraocular pressures were within normal limits. An anterior segment examination showed decreased tear film, but was otherwise unremarkable. A posterior segment examination showed bilateral CME with no vitreous cells. FA demonstrated bilateral petaloid leakage (Fig. 1) and CME was confirmed by OCT (Fig. 2). The CME was suspected to be secondary to risperidone and a recommendation about the possible association between the risperidone and macular edema was made to our patient’s psychiatrist, who decreased risperidone dosage from 3 to 2 mg/day when she followed up with them 2 months later. Her psychiatry team expressed concern with fully eliminating her risperidone or switching to another agent and risking a breakthrough psychotic episode. Thus, the psychiatrist recommended to first attempt dose reduction. At 4-months follow-up, her CME resolved bilaterally (Fig. 2) and vision improved to 20/40 in both eyes. OCT imaging 6 and 12 months after this visit showed no recurrence of CME. She has had no new ocular complaints since dosage adjustments as per record review, and no edema was noted on funduscopic examination at the last follow-up 18 months after presentation.

## Discussion and conclusions

CME has a broad differential diagnosis that includes surgical, vascular, structural, and medication-related causes such as from niacin [2] and E2 prostaglandins [1]. Multiple neurotrophic agents have adverse effects on the retina as well. Risperidone has effects on a variety of cellular receptors that may result in CME. These include a high affinity for serotonin receptors, a1-adrenergic and a2-adrenergic receptor blockade, and dopaminergic receptor blockade [5]. Research suggests that mechanisms such as vasorelaxation via alpha adrenergic blockade or direct effects on the retinal vascular endothelium may be responsible for risperidone-induced CME [6].

The Naranjo Probability Scale was used to assess the likelihood that the adverse drug reaction (CME) was due to the drug in question (risperidone) as opposed to other factors [4]. The probability results are classified as definite, probable, possible, or doubtful. We describe a second case in which a patient developed bilateral CME in association with risperidone and in our setting this was due to a “possible” adverse drug reaction linked to risperidone.

We note a limitation of our report is that our patient was concurrently using a topical prostaglandin analog and risperidone. Although the CME could have been secondary to the topical agent, the resolution of the CME occurred after the risperidone dosage was decreased while continuing the latanoprost. Another limitation is that our patient did not have the risperidone entirely discontinued as a precaution against breakthrough psychosis. She was stabilized on a lower dosage that has as of the last follow-up not resulted in recurrence of CME. Another limitation is that we elected not to retest at the higher dosage as her vision is stable and her psychiatry team is satisfied with current management. While the original report of Manousaridis and Gupta [3] showed resolution with risperidone elimination, we achieved a positive result with reduction. It is unclear whether the impact of risperidone on the development of CME is dose-dependent due to the scarcity of data regarding this effect and the rare occurrence of suspected risperidone-related macular edema. Further research would be of benefit.

The documentation of a second patient who developed CME associated with risperidone usage indicates that it may be beneficial to monitor patients taking risperidone for the development of maculopathy. In the first documented case, cessation of risperidone usage demonstrated a resolution of the patient’s condition. In our patient, resolution of CME was seen with a decrease in dosage. Awareness of the potential side effects of risperidone provides additional information to weigh in determining the most appropriate dosage and medication choice for patients requiring antipsychotic drugs.

## Abbreviations

CME: Cystoid macular edema
FA: Fluorescein angiography
OCT: Optical coherence tomography
